# Neonatal AKI profile using KDIGO guidelines: A cohort study in tertiary care hospital ICU of Lahore, Pakistan

**DOI:** 10.3389/fped.2022.1040077

**Published:** 2022-12-07

**Authors:** Rafia Gul, Zahid Anwar, Mehmood Sheikh, Ayesha Salamat, Samer Iqbal, Furqan Saleem, Samer Fatima

**Affiliations:** ^1^ Neonatal Intensive Care Unit, Fatima Memorial Hospital, Lahore, Pakistan; ^2^ Neonatal Intensive Care Unit, Jinnah Sindh Medical University, Karachi, Pakistan; ^3^ Department of Psychology, Ripah International University, Lahore, Pakistan

**Keywords:** AKI, sick neonates, survival, outborn, birth asphyxia, KDIGO

## Abstract

**Background and objective:**

Acute kidney injury (AKI) was observed in sick neonates and was associated with poor outcomes. Our cohort represents the neonatal characteristics of those diagnosed with AKI using Kidney Disease: Improved Global Outcome (KDIGO) guidelines.

**Methodology:**

A cohort study was conducted in the NICU of FMH from June 2019 to May 2021. Data were collected on a proforma. All continuous variables were not normally distributed and expressed as the median and interquartile range. Categorical variables were analyzed by proportional differences with the Pearson chi-square test or Fisher's exact tests. A multinomial logistic regression model was used to explore the independent risk factors for AKI. Time to the event (death) and the cohort's survival curves were plotted using the Cox proportional hazard model.

**Results:**

AKI occurred in 473 (37.6%) neonates. The risk factors of AKI were outborn birth [adjusted odds ratio (AOR): 3.987, 95% confidence interval (CI): 2.564–6.200, *p:* 0.000], birth asphyxia (AOR: 3.567, 95% CI: 2.093–6.080, *p*: 0.000), inotropic agent (AOR: 2.060, 95% CI: 1.436–2.957, *p*: 0.000), antenatal steroids (AOR: 1.721, 95% CI: 1.213–2.443, *p*: 0.002), central lines (AOR: 1.630, 95% CI: 1.155–2.298, *p*: 0.005) and intraventricular hemorrhage (IVH)/intracranial hemorrhage/disseminated intravascular coagulopathy (AOR: 1.580, 95% CI: 1.119–2.231, *p*: 0.009). AKI significantly increases the duration of stay and mortality rates by 16.5% vs. 3.9% in neonates with normal renal function (*p* < 0.001).

**Conclusion:**

About one-third of critically sick neonates had AKI. Significant risk factors for AKI were outborn birth, asphyxia inotropic agents, necrotizing enterocolitis, antenatal steroids central lines, and IVH. AKI is associated with an increased length of stay and increased mortality.

## Introduction

Acute kidney injury (AKI) is a condition characterized by an acute decline in kidney functions, affecting fluid and electrolyte homeostasis and the elimination of waste products (1). Neonates are at increased risk of acute kidney injury because of relatively immature kidneys and low glomerular filtration rate (GFR) (2, 3). In neonates, oliguria (urine output <0.5–1.0 ml/kg/h) may or may not be present in AKI (4). AKI has been reported in approximately one-third of critically ill neonates (5).

Multiple maternal and neonatal features act as risk factors for neonatal AKI. Maternal risk factors include pre-labor rupture of membranes (PROM), antepartum hemorrhage (APH), hypertension, antenatal steroids, and drug intake like nonsteroidal anti-inflammatory drugs (NSAIDs) during pregnancy. At the same time, neonatal factors include birth asphyxia, prematurity, congenital anomalies of the kidney and urinary tract (CAKUT), hemodynamic instability requiring inotropic support, sepsis, necrotizing enterocolitis (NEC), patent ductus arteriosus (PDA), nephrotoxic medications (like vancomycin and aminoglycosides), coagulopathy, and umbilical line placement (4, 6).

The severity, impact, and outcome assessment of neonatal AKI have always been challenging. A literature review shows that previously proposed models like the Acute Kidney Injury Network (AKIN) and Risk, Injury, and Failure; and Loss; and End-Stage kidney disease (RIFLE) have been used for diagnosing and categorizing the severity of neonatal AKI, but none is accepted universally. RIFLE, compared with Kidney Disease: Improved Global Outcome (KDIGO), has a disadvantage as it overestimates the incidence of AKI, particularly in stage 1, whereas AKIN underdiagnoses AKI, especially stage 3. The KDIGO classification has been proven to be a more reproducible evaluation tool with excellent prognostic abilities than the RIFLE or AKIN classification and has been specially modified for neonates. The KDIGO guidelines, specially modified for neonates, have been recently introduced for diagnosing and staging AKI (7).

International and national statistics regarding neonatal AKI using KDIGO guidelines are scarce. To bridge this gap, there is a need to conduct further studies. Our study aims to determine the profile of neonates with AKI, including prevalence, possible etiological factors, risk factors, and outcomes, using KDIGO guidelines in our tertiary care hospital ICU.

## Materials and methods

After ethical approval from the Institutional Review Board (IRB), a prospective cohort study was conducted. The study period spans 2 years, from June 2019 to May 2021, in the Department of Neonatology at Fatima Memorial Hospital, Lahore. We enrolled all neonates admitted to the NICU during the study period after seeking consent from their parents or guardians. However, all neonates with significant anomalies incompatible with life, congenital renal disorders, who did not survive until 72 h of postnatal life, or whose parents refused permission to participate in the study, were excluded. Serum creatinine was measured by cytometric analysis using the C311 Roche machine.

### Data collection

All neonates admitted to our NICU, fulfilling inclusion criteria with any clinical diagnosis, were enrolled in the study and managed as per departmental protocols. All admitted neonates were assessed for urine output (ml/kg/h) and serum creatinine.

All neonates' baseline serum creatinine level was measured within 24 h after enrollment in the study. Afterward, serum creatinine levels were checked every 24–48 h in case-specific conditions. The current study used serum creatinine levels to classify the degree of AKI following KDIGO guidelines. KDIGO diagnoses and classifies AKI in a tiered fashion using urine output and serum creatinine values as follows (7). However, AKI was never diagnosed in any neonate within the first 72 h of life.

• Stage 0: No change in S/Cr or rise <0.3 mg/dl or urine output ≥0.5 (ml/kg/h)death,
• Stage 1: S/Cr rise ≥0.3 mg/dl within 48 h or S/Cr rise ≥1.5–1.9 × baseline previous S/Cr or < 0.5 (ml/kg/h) for 6–12 h,
• Stage 2: S/Cr rise ≥2.0–2.9 times baseline previous S/Cr or <0.5 (ml/kg/h) for ≥12 h, and
• Stage 3: S/Cr rise ≥3.0 times baseline previous S/Cr or S/Cr ≥2.5 mg/dl (<10 ml/min/1.73 m^2^) or candidate for renal replacement therapy or <0.3 (ml/kg/h) for ≥24 h or anuria for ≥12 h.

Schwartz's formula (8) was used to calculate GFR as follows:

$$\mathrm{eGFR}=\frac{k\times \;\mathrm{length}\left(\mathrm{cm}\right)}{s\;\mathrm{Creatinine}\;\left(\mathrm{mg}/\mathrm{dl}\right)},$$

*k* = constant 0.33 for preterm and 0.45 for term babies.

The highest serum creatinine of neonates was used to calculate GFR.

Maternal demographic data was collected, including diabetes mellitus, hypertensive disorders, APH, parity, maternal anemia, antihypertensive drugs, and antenatal steroids during pregnancy. In addition, neonatal data include demographic characteristics like gestational age, weight, length, gender, and delivery mode. Clinical characteristics of neonates were mechanical ventilation (noninvasive/invasive), central line, and medications such as inotropic agents and nephrotoxic medications (vancomycin, aminoglycosides). Neonatal comorbid conditions included sepsis (leukocytosis or leukopenia or absolute neutrophilic count (ANC) < 1500 along with raised C-reactive protein (CRP) and platelets <100 and or positive blood culture), the presence of hemodynamically significant patent ductus arteriosus (hsPDA), dysnatremia (serum sodium level <135 or >145 mEq/lit), intraventricular hemorrhage (IVH), intracranial hemorrhage (ICH), disseminated intravascular coagulopathy (DIC), and NEC (modified Bell's stage II or III). All neonates were monitored until hospital discharge or death.

### Data analysis

Data were analyzed using the Statistical Package for the Social Sciences (SPSS 20.0). Descriptive statistics and tests of significance were calculated for all the variables. The Shapiro–Wilk test was used to assess the normality of the distribution of the investigated continuous parameters (length, weight, gestational age, and GFR). All continuous variables were not normally distributed and were expressed as the median and interquartile range (IQR). Categorical variables were analyzed by proportional differences with either the Pearson chi-square test or Fisher’s exact test. In addition, the z-test was applied to compare column proportions. The *p* < 0.05 was considered the level of significance unless adjusted by the Bonferroni method. All demographic data and clinical characteristics were compared between neonates with and without AKI. A multinomial logistic regression model was used to explore the independent risk factors for AKI. In addition, the time to the event (death) and the cohort’s survival curves were plotted using the Cox proportional hazard model.

## Results

**Figure 1:**
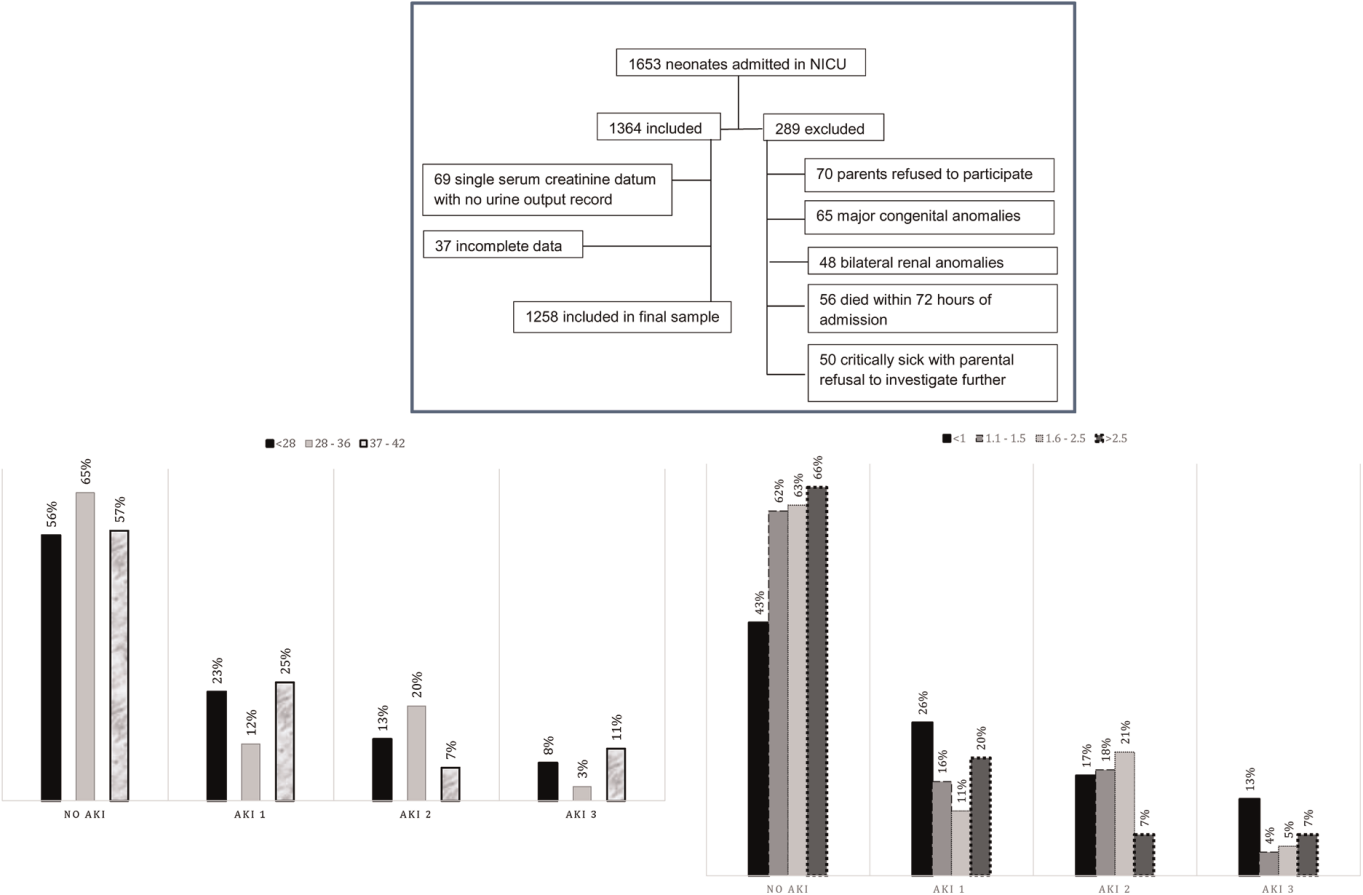
Consort flow chart of selected patients. Graph 1: Gestational age-based stratification of AKI. Graph 2: Neonatal weight-based stratification of AKI. AKI, acute kidney injury.

During the study period, 1,653 neonates were admitted to our neonatal intensive care units, out of which 289 were excluded. Of 1,364 neonates, 69 were excluded because of a single serum creatinine datum with no urine output record and 37 because of incomplete data. Therefore, a final analysis was performed on 1,258 neonates as shown in the consortium flow chart (Figure 1).

Gestational age- and weight-based stratification of AKI has been shown in graphs 1 and 2, respectively.

Of these 1,258 neonates, 473 (37.6%) had AKI, and among these, 197 (15.7%) had stage 1 AKI, 205 (16.3%) had stage 2, and only 71 (5.6%) had stage 3 AKI. The prevalence of different stages of AKI according to gestational age and weight has been shown in graphs 1 and 2, respectively.

On initial univariate analysis, maternal diabetes mellitus, maternal Hb > 10.5 mg/dl, antepartum hemorrhage, antihypertensive drugs, and antenatal steroids were risk factors for neonatal AKI. In addition, gestational age, birth asphyxia, mechanical ventilation, central lines, nephrotoxic drugs, inotropic agents, normal serum sodium levels, sepsis, NEC, IVH/ICH/DIC, hsPDA, and outborn birth were statistically significant neonatal risk factors for neonatal AKI. However, neonatal gender, birth order, cesarean section as the mode of delivery, and maternal history of hypertension or preeclampsia were statistically insignificant risk factors for neonatal AKI. Maternal and neonatal characteristics with and without AKI are shown in Table 1.

**Table 1:** Maternal and neonatal characteristics with and without AKI.

Parameters	No AKI (785)	Any stage AKI (473)	*p*-value
Neonatal characteristics			
Weight [median (IQR)]	2.2 (1.5–2.6)	2.2 (1.5–2.95)	0.004^a^
Gestational age [median (IQR)]	35 (33–36)	35 (33–37)	<0.001^a^
Length [median (IQR)]	43 (40–46)	43 (39–46)	0.053
Gender (male) [*n* (%)]	464 (59.1%)	259 (54.8%)	0.73
Gender (female) [*n* (%)]	321 (40.9%)	214 (45.2)	
Birth asphyxia [*n* (%)]	28 (3.6%)	53 (11.2%)	<0.001^a^
GFR [median (IQR)]	74.2 (47.8–131.6)	57.6 (32.5–71.5)	<0.001^a^
MV [*n* (%)]	396 (50.4%)	304 (64.3%)	<0.001^a^
Central lines [*n* (%)]	120 (15.3%)	146 (30.9%)	<0.001^a^
Nephrotoxic drugs [*n* (%)]	662 (84.3%)	368 (77.8%)	0.002^a^
Inotropic agents [*n* (%)]	103 (13.1%)	163 (34.5%)	<0.001^a^
Normal serum sodium level [*n* (%)]	59 (7.5%)	93 (19.7%)	<0.001^a^
Sepsis [*n* (%)]	310 (39.5%)	218 (46.1%)	0.013^a^
NEC [*n* (%)]	60 (7.6%)	95 (20.1%)	<0.001^a^
IVH/ICH/DIC [*n* (%)]	123 (15.7%)	105 (22.2%)	0.002^a^
hsPDA [*n* (%)]	111 (14.1%)	90 (19.0%)	0.014^a^
Out born [*n* (%)]	48 (6.1%)	121 (25.6%)	<0.001^a^
Maternal Characteristics			
LSCS [*n* (%)]	651 (82.9%)	379 (80.15)	0.120
Hypertension and eclampsia [*n* (%)]	184 (23.4%)	115 (24.3%)	0.387
Diabetes mellitus [*n* (%)]	152 (19.4%)	59 (12.5%)	0.001^a^
Maternal Hb > 10.5 g/l [*n* (%)]	72 (9.2%)	20 (4.2%)	0.001^a^
Antenatal steroids [*n* (%)]	630 (80.3%)	402 (85.0%)	0.020^a^
Antihypertensive drug [*n* (%)]	72 (9.2%)	20 (4.2%)	0.001^a^
Antepartum hemorrhage [*n* (%)]	38 (4.8%)	45 (9.5%)	0.001^a^
Primigravida [*n* (%)]	412 (52.5%)	227 (48.0%)	0.069

AKI, acute kidney injury; IQR, interquartile range; GFR, glomerular filtration rate; NEC, necrotizing enterocolitis; IVH, intraventricular hemorrhage; ICH, intracranial hemorrhage; DIC, disseminated intravascular coagulopathy; hsPDA, hemodynamically significant patent ductus arteriosus; LSCS, low segment caesarean section; MV, mechanical ventilation.

^a^ Statistically significant (p < 0.05).

All statistically significant risk factors in univariate analysis were further analyzed using multinominal logistic regression. These results show that the outborn neonates had a [adjusted odds ratio (AOR): 3.98] higher risk of AKI than the inborn. Birth asphyxia increased the risk of developing AKI (AOR: 3.567) followed by inotropic agents, by NEC, by antenatal steroids, by central lines by IVH/ICH/DIC, by sepsis, by hsPDA, and mechanical ventilation. All these factors were statistically significant (Table 2).

**Table 2:** Multinominal logistic regression for risk factors of AKI.

Risk factors	*p*-value	AOR	95% CI
IVH/ICH/DIC	0.009	1.580	1.119	2.231
Central lines	0.005	1.630	1.155	2.298
Inotropic agent	0.000	2.060	1.436	2.957
Antenatal steroids	0.002	1.721	1.213	2.443
Normal serum sodium level	0.038	0.515	0.275	0.963
Outborn	0.000	3.987	2.564	6.200
Birth asphyxia	0.000	3.567	2.093	6.080
Diabetes mellitus	0.000	0.436	0.298	0.638
Maternal Hb > 10.5 gm/dl	0.031	0.535	0.303	0.944

AKI, acute kidney injury; AOR, adjusted odds ratio; CI, confidence interval; IVH, intraventricular hemorrhage; ICH, intracranial hemorrhage; DIC, disseminated intravascular coagulopathy.

**Figure 2:**
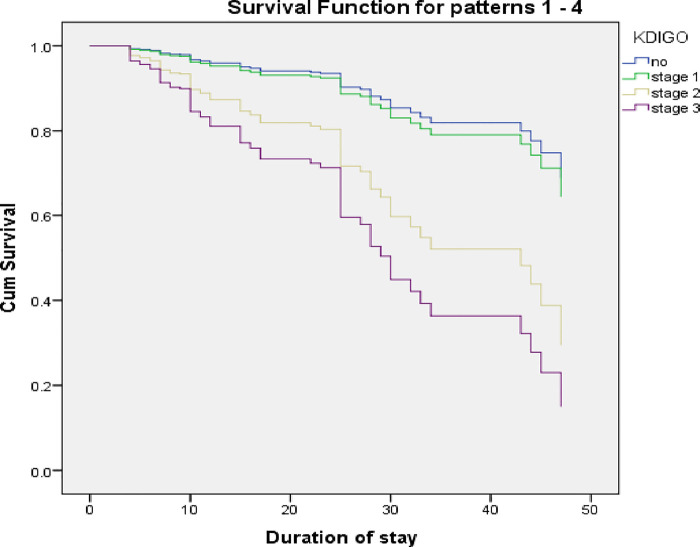
Survival curves.

Mortality rates were higher in neonates with AKI than in the non-AKI group. Of the 473 neonates with AKI, 16.5% expired, compared with only 3.9% of the 754 infants without AKI (*p* < 0.001). Among all neonates with acute kidney injury, neonates with AKI stage 3 had higher mortality rates (42.3%) as compared with stage 1 (8.1%) and stage 2 (15.6%), respectively. Similarly, the length of stay was directly related to the severity of AKI. The length of stay was longer in neonates with acute kidney injury (Table 3). The cohort’s survival curves were plotted using the Cox proportional hazard model (Figure 2). Neonates with AKI had compromised survival outcomes, most of whom expired within 30 days of their hospital stay (Figure 2).

**Table 3:** Duration of stay and clinical outcomes by AKI status.

Outcome	Any AKI			AKI (KDIGO staging)				
	No (785)	Yes (473)	*p*-value	0 (785)	1 (197)	2 (205)	3 (71)	*p*-value
Survived, *n* (%)	Yes	754 (96%)	395 (83%)	<0.001	754 (96%)	181 (92%)	173 (84%)	41 (58%)	<0.001
	No	31 (4%)	78 (17%)		31 (4%)	16 (8%)	32 (16%)	30 (42%)	
Length of stay, median (IQR)		7 (6–10)	9 (6–15)	<0.001	7 (6–10)	9 (6–16)	9 (7–15)	14 (7–21)	<0.001

AKI, acute kidney injury; KDIGO, Kidney Disease: Improved Global Outcome; IQR, interquartile range.

## Discussion

AKI is associated with a clinically significant outcome. However, only limited data is available regarding the diagnosis of AKI in neonates, using recently introduced KDIGO guidelines, specially modified for neonates.

AKI is common in sick neonates admitted to the NICU. Charlton et al. and Jetton et al. have reported the incidence of AKI in 21% and 30% of sick neonates admitted to NICU, respectively (5, 9). More than one-third of the neonates in our cohort had AKI. The similarity in findings is attributable to the resemblance in study settings, and all gestational ages were recruited.

No gestational age is exempt from AKI (5, 9–12). In preterm and low birth weight neonates, the prevalence of AKI has been reported as 56% by Lee et al., 63.3% by Lei et al., and 56.1% by Shalaby et al., respectively (13–15). Our preterms of <28 weeks had almost similar AKI incidence at 43.6%. Preterm neonates are vulnerable to AKI due to immature kidneys and nephrogenesis (16). According to our cohort, the AKI rate in neonates born at 37 weeks and above is consistent with that in other studies of term neonates (17–19).

All sick neonates transferred from other health facilities are at high risk of AKI (5, 9, 20, 21). Our study shows that all neonates who were outborn and referred to our tertiary level of care nursery had four times higher odds of AKI than inborn neonates. One possible explanation could be an association of comorbid conditions leading to renal damage and consequently requiring transfer to tertiary level care for ideal management.

The severity of renal failure is directly related to the severity of asphyxia. The incidence ranges from 9.1% to 56% with moderate to severe asphyxia (22). According to Gallo et al., Momtaz et al., and Elmas et al., 70%, 4%, and 20% of the study population with birth asphyxia developed AKI, respectively (23–25). Birth asphyxia is attributed to a decrease in renal blood flow and an increase in renal vascular resistance, which is contributed by acidosis, a high level of angiotensin II, prostaglandins, nitric oxide, and catecholamine (1, 19, 26).

Our cohort shows that neonates with birth asphyxia had a 256% higher risk of developing AKI. Neonates requiring inotropic support have a higher incidence of AKI, as reported in multiple studies (6, 10, 11, 25, 27). All of our neonates who required inotropic agents had double the odds of AKI. It has been speculated that shock requiring inotropic agents as *per se* is a promoter of AKI as it alters renal blood flow and starts a cascade of renal damage by vasoconstriction (10).

NEC potentiates an acute kidney insult. Hu et al. have shown that the risk of developing AKI increases six times in the presence of AKI (28). This association is attributed to an inflammatory cascade, a hallmark of NEC, that leads to a disturbance at the microcirculation level, leading to progressive afferent arteriolar vasoconstriction and continuous ultrafiltrate loss (29). However, in our study, NEC could not be proven to be a significant risk factor for AKI.

Central catheters are lifelines for neonates during critical periods. Elmas et al. (25) have observed a significant incidence of AKI in neonates with a central venous catheter (*p*: 0.001) and an umbilical catheter (*p*: 0.002). AlGadeeb et al. has documented that the presence of central lines and umbilical arterial catheters is significantly associated with AKI (27). In our cohort, all neonates with central lines had a significantly higher incidence of AKI. One possible explanation could be the combined damaging effect of central lines in the presence of other risk factors (30, 31).

AlGadeeb et al. and Marciniak et al. have reported intraventricular hemorrhage as a significant risk factor for neonatal acute kidney injury [AOR: 2.605, 95% confidence interval (CI): 1.465–4.631, *p* < 0.001] and (OR: 2.38, 95% CI: 1.46–3.87) respectively (27, 32). DIC has been reported as a significant risk factor for neonatal acute kidney injury by Nickavar et al. Our study highlights the association between intraventricular hemorrhage and disseminated intravascular coagulation with acute renal insult. DIC and IVH decrease adequate circulatory volume, resulting in renal hypo-perfusion and failure (3).

Gestational diabetes mellitus (GDM) is not an additional risk factor for neonatal renal insult (5, 10, 13). However, Cappuccini et al. have demonstrated that hyperglycemia negatively impacts developing fetal kidneys, resulting in reduced nephrogenesis, tubular integration, and renal failure (33). However, Aisa et al. have found that the neonates of mothers who maintained strict glycemic control during pregnancy and fulfilled the other criteria of the GDM management program showed no differences compared with the normoglycemic group (34). In our study, maternal diabetes mellitus instead appears to have a protective role against neonatal AKI. One possible explanation might be that during the antenatal period, these mothers have reasonable glycemic control, vigilant monitoring, and regular checkup hence optimal fetal nephrogenesis.

The optimal fetal growth and development are coupled with optimal maternal health status. Maternal anemia is associated with adverse neonatal outcomes (5, 9, 12). Our study population showed that all neonates of non-anemic mothers were protected from acute kidney injury. One possible explanation could be the impact of maternal hemoglobin status on the fetal metabolic signaling pathway affecting “fetal programming” (35).

Dysnatremia, either hyponatremia or hypernatremia, places neonates at risk of developing AKI. Hyponatremia was more prevalent in neonates with AKI than neonates without AKI (36).

Hypernatremia has been reported as a significant risk factor for AKI by Hamsa and Farhad (36–38). However, Basalely et al. has reported that dysnatremia was not associated with AKI (38). Neonates with normal sodium levels have 48.5% less risk of developing AKI. Dysnatremia is associated with volume depletion and shock. Hence, it leads to decreased renal blood flow resulting in renal vasoconstriction, decreased GFR, and ultimately causes renal failure (2).

Mechanical ventilation, hsPDA, and sepsis are considered significant risk factors for neonatal AKI (1, 19, 23–28). Our study shows that these factors add risk to renal insult but are statistically insignificant. One possible explanation could be the homogeneous presence of these factors in all neonates of the cohort. Vancomycin and amikacin are nephrotoxic drugs and cause AKI (1, 19, 39). However, the meta-analysis by Hu et al. shows that a combination of these medications did not aggravate harmful effects on kidneys (28). Similar results have been reflected in our study that shows that these drugs have a negligible contribution to AKI.

The supporting data from other studies highlight that AKI is associated with poor outcomes (1–13). Analyses by Jetton et al. show that neonates with AKI have 8.8 times longer duration of stay and 4.6 times higher mortality compared with the non-AKI group (5). When comparing different stages of AKI, the higher the order of renal failure, the poorer is the outcome (11, 20, 21, 26, 27). Irrespective of severity, all neonates in our cohort with AKI had a significantly longer length of stay and higher odds of death. AKI, in the presence of other comorbid conditions, requires medical treatment, prolongs hospital stay, and is responsible for poor neonatal outcomes.

## Conclusion

AKI has been reported in approximately one-third of critically ill neonate according to our study. The most significant risk factors were outborn birth, birth asphyxia, and inotropic agents, followed by antenatal steroids, central lines, and IVH/ICH/DIC. AKI not only prolongs the duration of stay but also reduces the survival of sick neonates.

### Limitations

There are several limitations to this study. First, only serum creatinine level was used to diagnose and stage renal failure, and urine output was not incorporated. Urine output monitoring by catheterization was never planned to avoid catheter-associated urinary tract infections. Pamper weight method was also not selected because of the frequent presence of stool with urine during pampering. Second, the long-term outcome was neither planned nor monitored. Third, the higher incidence of AKI would have been expected if it had included all neonates with bilateral renal anomalies, significant congenital anomalies, and criticality sick neonates who were first enrolled and then excluded due to parental refusal. Another limitation is the temporal problem related to some of the variables like NEC (as described above) making the direction of the association more difficult to determine. This study did not include all possible risk factors of neonatal AKI, including bilirubin level.

## Data Availability

The raw data supporting the conclusions of this article will be made available by the authors, without undue reservation.
